# Supplementary material for: Ni-Catalyzed Enantioselective Intramolecular Mizoroki–Heck Reaction for the Synthesis of Phenanthridinone Derivatives
Source: J Org Chem. 2023 Jun 15;88(13):8203–26. doi: 10.1021/acs.joc.3c00202 (PMC10337041; doi:10.1021/acs.joc.3c00202)

dr-112-096-f10-12 DMSO 350 K
PROTON DMSO {D:\nmrusers\malachowski} BM 7

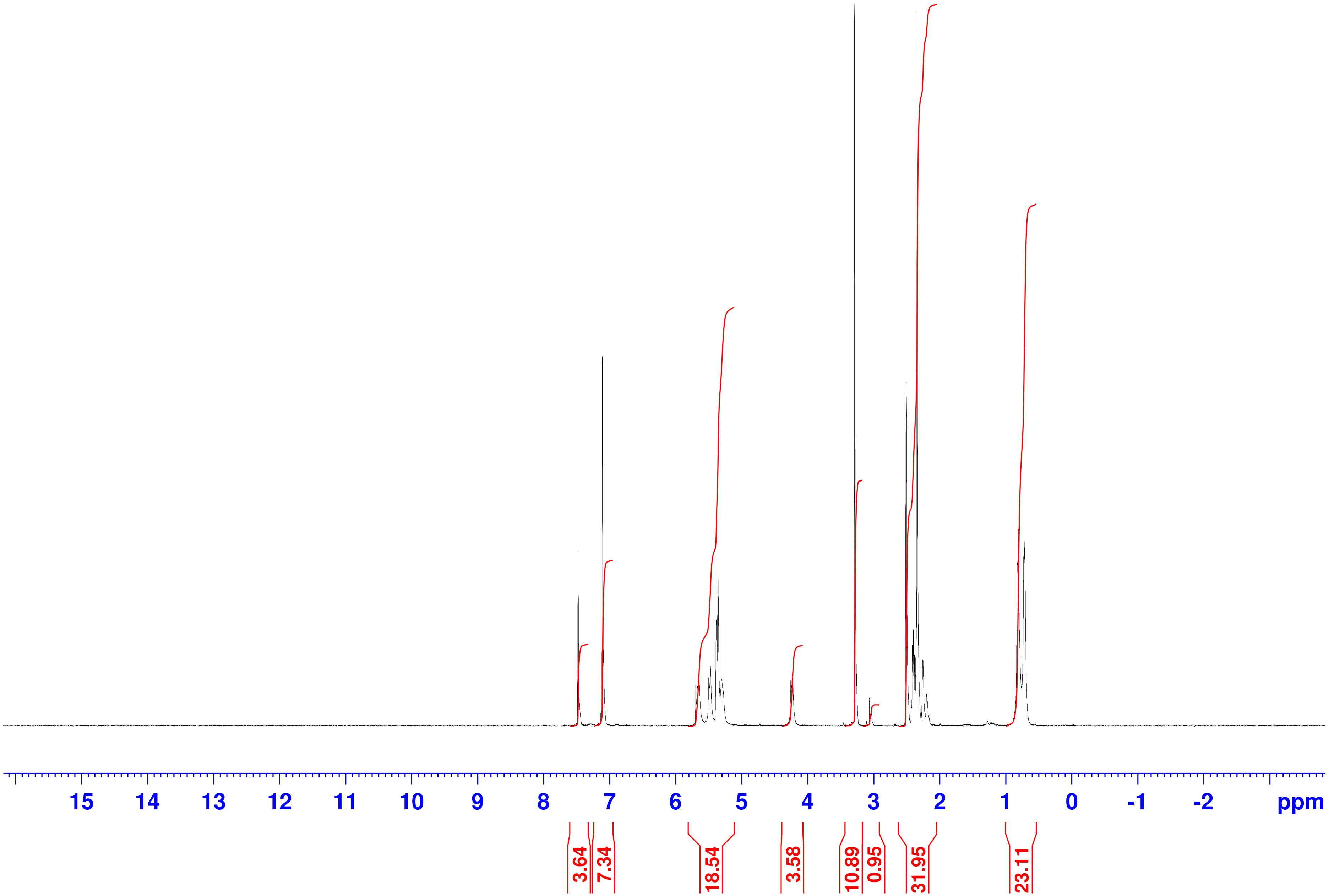

Supplement: Supplementary file 2 — jo3c00202_si_002.zip [file jo3c00202_si_002.zip › Amides (N-MOM)/1k iPr-NMOM-4Me-ArBr/1H iPr-NMOM-4Me-ArBr 1k/1/pdata/1/email_dr-112-096-f10-12 DMSO 350K_1_1.pdf]
